# Eating Disorder Awareness Campaigns: Thematic and Quantitative Analysis Using Twitter

**DOI:** 10.2196/17626

**Published:** 2020-07-14

**Authors:** Iranzu Viguria, Miguel Angel Alvarez-Mon, Maria Llavero-Valero, Angel Asunsolo del Barco, Felipe Ortuño, Melchor Alvarez-Mon

**Affiliations:** 1 Department of Psychiatry and Medical Psychology Clinica Universidad de Navarra Pamplona Spain; 2 Department of Medicine and Medical Specialities University of Alcala Alcalá de Henares Spain; 3 Department of Psychiatry and Medical Psychology Hospital Universitario Infanta Leonor Madrid Spain; 4 Department of Endocrinology and Nutrition Clinica Universidad de Navarra Pamplona Spain; 5 Department of Surgery, Medical and Social Sciences University of Alcala Madrid Spain; 6 Internal Medicine and Immune System Diseases-Rheumatology Service University Hospital Príncipe de Asturias Alcala de Henares Spain; 7 Centro de Investigación Biomédica en Red de Enfermedades Hepáticas y Digestivas Instituto Ramón y Cajal de Investigaciones Sanitarias Madrid Spain

**Keywords:** awareness campaigns, eating disorders, Twitter, social media

## Abstract

**Background:**

Health awareness initiatives are frequent but their efficacy is a matter of controversy. We have investigated the effect of the Eating Disorder Awareness Week and Wake Up Weight Watchers campaigns on Twitter.

**Objective:**

We aimed to examine whether the Eating Disorder Awareness Week and Wake Up Weight Watchers initiatives increased the volume and dissemination of Twitter conversations related to eating disorders and investigate what content generates the most interest on Twitter.

**Methods:**

Over a period of 12 consecutive days in 2018, we collected tweets containing the hashtag #wakeupweightwatchers and hashtags related to Eating Disorder Awareness Week (#eatingdisorderawarenessweek, #eatingdisorderawareness, or #EDAW), with the hashtag #eatingdisorder as a control. The content of each tweet was rated as medical, testimony, help offer, awareness, pro-ana, or anti-ana. We analyzed the number of retweets and favorites generated, as well as the potential reach and impact of the hashtags and the characteristics of contributors.

**Results:**

The number of #wakeupweightwatchers tweets was higher than that of Eating Disorder Awareness Week and #eatingdisorder tweets (3900, 2056, and 1057, respectively). The content of tweets was significantly different between the hashtags analyzed (*P*<.001). Medical content was lower in the awareness campaigns. Awareness and help offer content were lower in #wakeupweightwatchers tweets. Retweet and favorite ratios were highest in #wakeupweightwatchers tweets. Eating Disorder Awareness Week achieved the highest impact, and very influential contributors participated.

**Conclusions:**

Both awareness campaigns effectively promoted tweeting about eating disorders. The majority of tweets did not promote any specific preventive or help-seeking behaviors.

## Introduction

Eating disorders belong to a wide group of disorders characterized by weight concern, body image preoccupation, and persistent disturbances in eating that impair health and psychosocial functioning [1]. These diseases occur worldwide, mostly commonly in young women, with an estimated lifetime prevalence of 0.8% for anorexia nervosa and binge eating disorder and 0.3% for bulimia nervosa [2,3]. The etiology of these disorders is complex and not fully understood. Nonetheless, many aspects of Western culture, such as the “thin ideal” or media pressure, promote an obsession with weight loss and body image [4].

Several strategies have been established to reduce the prevalence of eating disorders [5]. Health awareness initiatives have become a frequent intervention strategy, as high-visibility programs have stimulated the discussion of certain health risks and enhanced public exposure to these issues [6]. For instance, Eating Disorder Awareness Week is an annual event sponsored by a wide range of partners that aims to increase public recognition of the preventive and supportive options for individuals suffering from eating disorder behaviors [7]. However, some initiatives are not scheduled in advance, as was the case with Wake Up Weight Watchers. On February 6, 2018, many health care providers were concerned when Weight Watchers announced free memberships for teenagers, because dieting as a teenager increases the risk of developing eating disorders and risky behaviors [8,9]. Thus, the Wake Up Weight Watchers initiative was developed to counteract the potentially negative effects of adolescent dieting [10]. Although the efficacy of health awareness initiatives is a matter of controversy, analytical studies of those related to eating disorders are particularly scarce [11].

Notably**,** research using Twitter has been an effective assessment strategy for analyzing patient attitudes toward various medical topics, including mental health [12,13]. Additionally, Twitter has been used to assess the impact of disease awareness programs on users, especially young adults and adolescents [14]. Thus, this platform could help health care providers effectively reach many young people troubled by eating disorders [15]. It has been shown that medical interventions conducted through Twitter can modify health-related behaviors [16]. Therefore, this platform could provide a good setting for the promotion of healthy lifestyles among at-risk individuals and encourage them to get in touch with health providers [17]. Previous research has found that engaging in help-seeking behavior has been an important factor in protecting the mental health of young people [18]**.** Despite these efforts to help, evidence nevertheless suggests that those experiencing eating disorder thoughts and behaviors are less likely to seek help for their mental health difficulties [19]. One recent study even reported that body image was the second leading personal concern for which people between the ages of 18 and 25 years sought help online [20].

The aims of this study are the following: (1) Examine the volume of Twitter conversations related to eating disorders generated by the Eating Disorder Awareness Week and Wake Up Weight Watchers initiatives, and compare the dissemination of these tweets with tweets related to eating disorders that did not use the official hashtags of these campaigns. (2) Describe the main content themes of tweets from both initiatives and compare them to those of typical tweets related to eating disorders. (3) Investigate what content generates the most interest on Twitter. (4) Identify and categorize the most active and influential users among those that participate in these conversations.

## Methods

### Research Strategy

In this observational quantitative and qualitative study, we focused on searching for tweets that referred to two eating disorder campaigns in particular: Eating Disorder Awareness Week (#eatingdisorderawarenessweek, #EDAW, #eatingdisorderawareness) and Wake Up Weight Watchers (#wakeupweightwatchers). As a control, we simultaneously studied those tweets related to eating disorders stemming from the hashtag with the name of the disease (#eatingdisorder). The inclusion criteria for tweets were the following: (1) tweeted by a public account, (2) included the previously mentioned hashtags, (3) posted between February 24 and March 7, 2018, and (4) posted in English. The 12-day period was chosen to align our research with the dates of the two campaigns included in this study. Eating Disorder Awareness Week 2018 started February 26, 2018, and the Wake Up Weight Watchers initiative began in February 2018. In addition, we obtained the number of retweets and favorites each tweet generated, the date and time of each tweet, the potential reach and impact of each hashtag, as well as the user’s profile description.

### Search Tool and Data Collection

For this study, we used the Twitter Firehose (Gnip) data stream, which allows access to 100% of all public tweets that match a certain criteria (query) [21]. In our study, the search criteria were the previously mentioned hashtags. Tweet Binder, the search engine we employed, uses automatic machine learning text analysis algorithms, as well as node.js and PHP language; this enabled us to analyze tweets in a JavaScript Object Notation (JSON) format, which is used by Gnip.

### Content Analysis Process

All 7468 retrieved tweets were directly inspected by two raters (authors IV and MAAM). First, we scanned all of the tweets, and excluded 455 tweets that provided information that was too limited (ie, tweets consisting mainly of hashtags), contained only pictures, or included hashtags of both initiatives. All remaining tweets were considered for content analysis. Second, we created a codebook based on our research questions, our previous experience in analyzing tweets, and what we determined to be the most common tweet themes. Third, IV and MAAM analyzed 300 tweets separately to test the suitability of the codebook. Discrepancies were discussed between the raters and with another author (MLV). After revising the codebook, the interrater reliability was reassessed with a different set of 159 tweets. As this resulted in adequate κ values (range 0.89-0.99), the raters then proceeded to perform a content analysis of 1500 tweets, including 500 tweets each from the three different groups of hashtags, randomly selected. Each tweet, depending on the content, was rated as medical, personal testimony, pro-ana (tweets glorifying or encouraging ED behaviors or portraying them as a lifestyle choice), anti-ana (tweets confronting pro-ana ideas), awareness, or help offer. The coding categories were not mutually exclusive. If the contents of a tweet were repeated exactly or almost identically in other tweets, they were classified in the same way as the first tweet encountered. The classification criteria we used and examples of tweets according to category are shown in Table 1.

**Table 1:** Category definitions and examples of classification^a^.

Categories and definitions	Examples of classification
Medical (clinical or epidemiological information)	“For #EatingDisorderAwarenessWeek remember that1) ppl with eating difficulties are not always underweight 2) it’s not about wanting to diet and be thin 3) recovery is not as simple as “eat a bit more to recover” 4) ED’s are life threatening illnesses”
Testimony (expression of experience)	“When I was about to become a teenager, my parents put me on Weight Watchers. I have been battling Anorexia since then. Dieting is one of the most common antecedents of disordered eating in young people. #WakeUpWeightWatchers” “We are warriors whether we are still battling this war or we have overcome and conquered it. We stand together in a battle which costs lives and relationships but we stand together. #EatingDisorderAwarenessWeek” “My younger sister has this too - as if she is cured and we can now resume normal life and carry on from where we left off. But everything has changed. #StateNotWeight #EatingDisorderAwarenessWeek” “As someone who suffered from an ED after dieting I am so worried about promoting weight watchers to teenagers. It may be dangerous. #WakeUpWeightWatchers”
Pro-ana (pro–eating disorder content, promoting compensatory behaviors or disordered eating for weight loss)	“Ohh wow my dad really bought me the scale I wanted. I'm so excited but at the same time it's better he doesn't know why I really wanted it so bad. #eatingdisorder #proana #ed #rexi” “I can’t eat because I’m allergic to calories. #ana #mia #anorexia #anorexic #anoressia #anoressica #anorexie #anorexique #vita #lavie #mystory #Mylife #conversation #quote #quotes #meme #memes #MEMES #anamia #proana #eatingdisorder #diary #mydiary #me #life” “Skip dinner, wake up thinner. #Eatingdisorder. 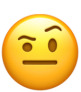 ” “Just think how all of this pain will be worth it in the end #thinspo #thin #skinny #eatingdisorder #anorexic #anorexia #mia”
Anti-ana (expressing concerns regarding pro-ana content)	“As the mom of a teen who struggles with an eating disorder, seeing WW target teens makes me worry. It is not correct to tell kids that a number on the scale is what gives them their value. #livelife #smile #behappy #BodyPositivity #WakeUpWeightWatchers”
Help offer (promoting help- and treatment-seeking behaviors)	“It's #EatingDisorderAwarenessWeek If you have concerns about your loved ones or if you feel keen to talk to a professional counselor about your difficulties, call our confidential helpline at XXX #EDAW18 #NotAlone #HeretoHelp” “Are you concerned that your child may have an #eatingdisorder? Help and support is available from @XXX”
Awareness (aim to raise awareness, combat misconceptions, or educate)	“Sign this petition against @WeightWatchers' insidious campaign to recruit teens to their dangerous business #WakeUpWeightWatchers” “Eating disorder experts, dietitians, physicians, and advocates around the world are standing together against @weightwatchers marketing dangerous “free” summer memberships to vulnerable young people. A season of fat-shaming, a lifetime of weight cycling.” “Spotting the signs: “If you suspect an eating disorder you should talk to them directly, and express your concerns –because the sooner you get help the better“ #EatingDisorderAwarenessWeek #SpeakYourMind”

^a^Usernames and personal names have been removed.

### Measuring Influence on Twitter: Retweets, Favorites, and the Reach and Impact of Hashtags

We analyzed the number of retweets and favorites generated by each tweet as an indicator of user interest in a given topic. We also measured the potential reach and impact of all analyzed hashtags to best assess tendencies in the dissemination of tweets. For the purpose of this study, impact is defined as a numerical value representing the potential views a tweet may receive, while reach is defined as a numerical value measuring the potential audience of the hashtag. These metrics are analyzed by Tweet Binder, a Twitter tool that automatically tracks hashtags. A more detailed explanation is shown in Multimedia Appendix 1.

### User Categorization

The number of tweets that each Twitter user (contributor) posted with a certain hashtag was automatically quantified by Tweet Binder. We defined the contributors as very active if posting at least 6 tweets with a certain hashtag. The number of followers of the contributors was automatically quantified by Tweet Binder at the time of the study. We classified those with at least 5000 followers at the time of our analysis as very influential contributors. Furthermore, we collected the profile description of the ten most influential and active users that participated in these conversations. The user profile description was used to determine whether a tweet was posted by health providers (such as physicians or nurses), health organizations (hospitals, journals, medical societies, or research institution), or by non–health-related users.

### Ethical Considerations

This study was approved by the Research Ethics Committee of University of Navarra. This study did not directly involve human subjects and did not include interventions, but instead used publicly available tweets. Nevertheless, we have taken care to not reveal any username and to avoid citing the tweets that could reveal usernames.

### Statistical Analysis

A descriptive study of the sample was performed, describing the variables by their absolute and relative frequencies. The percentages found were compared using the chi-square test. The mean and median numbers of retweets or favorites per original tweet about the different hashtags studied were compared using the Kruskal-Wallis test. All statistical analyses were performed using SPSS (Version 20, IBM Corp).

## Results

### Public Awareness Initiatives Succeeded in Spreading Their Tweets

Our search tool found that the total number of tweets generated with the hashtags associated with the Eating Disorder Awareness Week and Wake Up Weight Watchers awareness campaigns and the control hashtag #eatingdisorder were 7468**.** Of these, 455 were excluded according to the criteria of the study. The number of #wakeupweightwatchers tweets (3900) was double that of the Eating Disorder Awareness Week tweets (2056 tweets included the hashtags #eatingdisorderawarenessweek, #eatingdisorderawareness, or #EDAW). There were 1057 tweets using the control hashtag #eatingdisorder.

As shown in Table 2, we found that the potential impact and reach of the tweets was higher among Eating Disorder Awareness Week hashtags (54,143,739 and 9,547,326, respectively) and #wakeupweightwatchers (28,371,411 and 9,332,345, respectively) than observed with the #eatingdisorder hashtag (7,384,797 and 4,175,017, respectively).

**Table 2:** Potential impact, potential reach, number of contributors, percentage of very active users, and percentage of very influential users in the 3 groups.

Parameters	Eating disorder	Eating Disorder Awareness Week	Wake Up Weight Watchers
Potential impact, n	7,384,797	54,143,739	28,371,411
Potential reach, n	4,175,017	9,547,326	9,332,345
Contributors, n	1243	2531	4609
Very active contributors, n (%)	70 (5.6)	59 (2.3)	383 (8.3)
Very influential contributors, n (%)	127 (10.2)	186 (7.4)	289 (6.3)

### Eating Disorder Awareness Campaigns Fail to Promote Medical Content, Help Offers, Or Personal Testimonies But Can Increase Pro-Ana Criticism

**Figure 1:**
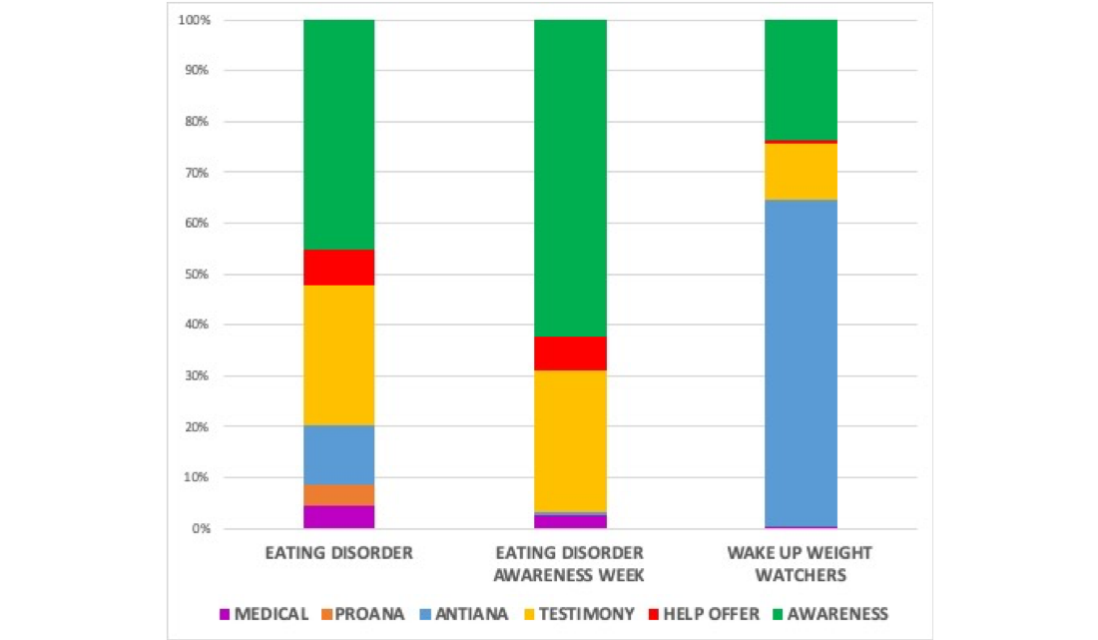
Different percentages of tweets in each category generated about the awareness campaigns #eatingdisorderawarenessweek (including #eatingdisorderawareness and #EDAW) and #wakeupweightwatchers, with #eatingdisorder as the control. Percentages were calculated with respect to the 500 tweets analyzed for each of the three categories of hashtags. Chi-square test; *P*<.001.

We performed a content analysis of 1500 tweets, including 500 randomly selected tweets each from the three different groups of hashtags. First, we studied the medical contents of the tweets. Although the frequency was low, there were significant differences between the 3 groups (*P*<.001; Figure 1). The percentage of tweets with medical content was higher in those with the #eatingdisorder hashtag than in those with the awareness campaign hashtags*.* The lowest frequency was found in the #wakeupweightwatchers group.

We also analyzed the content of the 1500 tweets according to the other five established categories: personal testimony, help offer, awareness, pro-ana, and anti-ana. Different patterns of distribution among the five categories were found between the 3 groups of hashtags (*P*<.001). The percentage of tweets with personal testimony content was similar in #eatingdisorder tweets and tweets using Eating Disorder Awareness Week hashtags, while the percentage was lower in #wakeupweightwatchers tweets (Figure 1). The frequency of awareness tweets was higher in the tweets with Eating Disorder Awareness Week hashtags and lower in the tweets with the #wakeupweightwatchers hashtag (Figure 1). Help offer tweets were more prevalent in tweets with the #eatingdisorder hashtag and Eating Disorder Awareness Week hashtags. The frequency of anti-ana tweets was clearly higher in the #wakeupweightwatchers group and minimal in Eating Disorder Awareness Week tweets (Figure 1). Pro-ana content was absent in #wakeupweightwatchers tweets and the frequency was highest in #eatingdisorder tweets (Figure 1).

### Pro-Ana and Medical Contents Did Not Attract Much Attention Among Twitter Users

We measured the number of retweets and favorites generated by each hashtag and category. Since these hashtags have different contribution numbers, we used ratios, dividing by the total number of tweets in each campaign. We found that the retweet-to-tweet and favorite-to-tweet ratio between the three hashtag groups were significantly different (*P*<.001; Table 3). As whole group, #wakeupweightwatchers was the hashtag with the highest retweet-to-tweet and favorite-to-tweet ratio. We found that the retweet-to-tweet and favorite-to-tweet ratio between the six categories were significantly different (*P*<.05; Table 4). Regarding the medical content, the highest ratios were found in #wakeupweightwatchers tweets, while they were low among the #eatingdisorder and Eating Disorder Awareness Week hashtags. Furthermore, tweets with personal testimony, help offer, and awareness content showed higher retweet-to-tweet and favorite-to-tweet ratio in #wakeupweightwatchers than those found in the other two groups. The tweets with anti-ana content generated higher ratios in #eatingdisorder tweets and lower ratios in Eating Disorder Awareness Week tweets. We found that the pro-ana tweets generated no or low numbers of retweets and likes in the 3 groups of tweets studied.

**Table 3:** Retweet-to-tweet and favorite-to-tweet ratio per hashtag analyzed.

Parameter and value		Eating disorder		Eating Disorder Awareness Week		Wake Up Weight Watchers	*P* value^a^	
**Retweet**								
	Mean (SD)		0.84 (7.21)		0.1 (0.79)	4.23 (21.51)		N/A^b^
	Median (IQR)		0 (0-0)		0 (0-0)	1 (0-3)		<.001
**Favorite**								
	Mean (SD)		1.76 (16.46)		0.2 (1.43)	9.52 (58.09)		N/A
	Median (IQR)		0 (0-0)		0 (0-0)	2 (0-7)		<.001

^a^The Kruskal-Wallis test was used.

^b^N/A: not applicable.

**Table 4:** Retweet-to-tweet and favorite-to-tweet ratio per category in each hashtag analyzed.

Category and parameter		Eating disorder, median (IQR)	Eating Disorder Awareness Week, median (IQR)	Wake Up Weight Watchers, median (IQR)	*P* value^a^
**Medical**					
	Retweet	0 (0-0)	0 (0-0)	4 (3)	.03
	Favorite	0 (0-1)	0 (0-0)	4 (1-4)	.02
**Pro-ana**					
	Retweet	0 (0-0)	0 (0-0)	—^b^	.88
	Favorite	0 (0-1.75)	0 (0-0)	—	.72
**Anti-ana**					
	Retweet	2 (0-7.5)	0 (0-0)	1 (0-3)	.002
	Favorite	5 (1.5-9.5)	0 (0-0)	3 (1-7)	.001
**Personal testimony**					
	Retweet	0 (0-0)	0 (0-0)	1 (0-2)	<.001
	Favorite	0 (0-2)	0 (0-0)	3 (2-6.25)	<.001
**Help offer**					
	Retweet	0 (0-1)	0 (0-0)	2 (1.25-2)	.002
	Favorite	0 (0-1)	0 (0-1)	4 (0.5-6.75)	.047
**Awareness**					
	Retweet	0 (0-1)	0 (0-0)	1 (0-3)	<.001
	Favorite	0 (0-2)	0 (0-0)	3 (1-6.75)	<.001

^a^The Kruskal-Wallis test was used.

^b^Not available.

### Eating Disorder Awareness Campaigns Increased the Participation of Twitter Users, But Not of Those Considered Very Active or Influential

We also analyzed the number and type of users that participated in conversations using any of the three hashtag groups (Table 2). The total number of contributors was higher in the #wakeupweightwatchers group (4609) than in the Eating Disorder Awareness Week (2531) and #eatingdisorder groups (1243). The frequency of very active users was higher in the #wakeupweightwatchers group (8.3%) than in the #eatingdisorder (5.63%) and Eating Disorder Awareness Week groups (2.33%). The percentage of very influential participants was highest in #eatingdisorder group (10.22%) than in the #wakeupweightwatchers (6.27%) and Eating Disorder Awareness Week groups (7.35%). The percentage of health providers or health organizations among the ten most active or influential users that participated in these conversations was higher in the #wakeupweightwatchers group (40%) than in the #eatingdisorder (30%) and Eating Disorder Awareness (20%) groups.

Finally, we analyzed the 5 hashtags more frequently associated with the 3 groups of hashtags studied (Table 5). We found that the hashtags most frequently associated with the 3 groups analyzed were different.

**Table 5:** Top 5 hashtags associated with the 3 groups of hashtags analyzed.

Hashtag group	The top 5 hashtags and the number of tweets per hashtag
Eating disorder	Anorexia (n=199), HAES (Health At Every Size; n=195), HelpfaithHeal (n=193), bodypositive (n=171), recovery (n=156)
Eating Disorder Awareness Week	GlobalGoodEmi (n=1201), Winsday (n=289), Wednesday (n=287), Giveaway (n=196), Flash (n=180)
Wake Up Weight Watchers	HAES (n=341), simplyNYC (m=283), MNYFashionweek (n=281), sheopenedthedoor (n=281), weightwatchers (n=198)

## Discussion

### Principal Findings

In this study, we have demonstrated that the eating disorder awareness campaign initiatives Eating Disorder Awareness Week and Wake Up Weight Watchers generated a greater number of tweets, with more far-reaching dissemination than the control eating disorder hashtag. Nonetheless, they failed to increase the frequency of tweets containing medical content, personal testimonies of recovery, or offers for treatment. Moreover, those Twitter users classified as “very influential” did not command a strong presence in driving awareness of the aforementioned initiatives; on the whole, medical institutions also did not have a strong presence. Lastly, hashtags commonly used by pro-ana social networks are not used to any great extent by the awareness campaigns studied, thus making the campaigns less likely to reach at-risk individuals.

Our findings show that awareness initiatives generated a greater number of eating disorder–related tweets than those spontaneously posted. This finding suggests that awareness initiatives trigger conversations and social responses to eating disorders that would not have occurred otherwise. The total number of tweets related to the 3 groups of hashtags we analyzed over a period of 12 days was higher than the numbers of tweets associated with other mental health conditions [22]. However, the interest in eating disorders shown by the American media overall can be classified as low [23]. Notwithstanding, our results show that the Eating Disorder Awareness Week and Wake Up Weight Watchers campaigns had a positive effect on Twitter users. Furthermore, the potential impact and reach of the tweets related to the two campaigns were clearly higher than those achieved by our eating disorder control group.

According to the analysis of the generated tweets, both awareness initiatives have limitations. They failed to increase the percentage of tweets related to medical content, in contrast to previous evidence showing that tweets with medical content generated special interest among Twitter users [24]. A study performed within a population of young people highlighted the use of scientific data as being a key indicator of credibility in online resources [20]. The scarcity of medical content found by our study has also been reported in other awareness initiatives focused on other medical diseases, such as several prevalent types of cancer [25]. For example, Breast Cancer Awareness Month tweets most commonly contained content related to the practices of wearing pink, promoting walks/runs, and promoting fundraisers, as opposed to direct medical advice and preventive measures [25]. Furthermore, the frequency of tweets generated with help content was not higher in tweets related to awareness campaign hashtags. These results are concerning due to the existence of evidence that interventions that included help resources increased participants’ knowledge of treatment resources [7].

Interestingly, one-third of the tweets analyzed have personal testimonies. This finding supports the value of Twitter as a means of communicating personal content related to eating disorder symptoms [26,27]. One-third of US teenagers use Twitter and this share has remained consistent over the past several years [28]. In general, Twitter users tend to be younger, have more education, and have higher incomes than the overall US population, but these differences are minimal among teenagers [29]. In addition, similar results have been reported in other countries. Twitter is a predominantly public-facing platform. It gives anonymity to user testimonies, encourages communication by people with real or perceived personal or social restrictions, and prevents the potential stigmatization of those with mental health diseases [30]. In addition, it provides an insight into the potential value of Twitter as a tool to reach these patients and encourage them to get in touch with health care providers. Unfortunately, neither campaign increased the percentage of tweets that included testimonies. It has been shown that young people value resources that allow them to access the personal stories of peers with lived experiences [31]. As expected, Eating Disorder Awareness Week had the highest proportion of awareness tweets. There is no evidence that this type of content increases the odds of seeking help or changing behaviors.

The awareness initiatives increased the frequency of tweets with anti-ana content. Additionally, pro-ana contributors were absent. The analysis of the eating disorder tweets showed a very low percentage of pro-ana content. Several reasons that are not mutually exclusive may explain this finding. First, Twitter has a policy of excluding pro-ana content [32]. Second, the pro-ana community may communicate through other web-based means, such as pro-ana websites [33]. Third, pro-ana social media posts are often tagged using specific hashtags, such as #thinspo, #thinspiration, #Thin15, #edprobs, and #proana. Pro-ana communities on social media have been described as tighter and more cohesive than others, as well as more secretive and exclusive [34]. Our findings show that these pro-ana hashtags have not been included in tweets related to the awareness campaigns or tweets tagged with #eatingdisorder. These findings suggest that Twitter awareness campaigns should include hashtags commonly used by these pro-ana communities to increase the likelihood of reaching people in need of interventions for disordered eating behaviors [35,36].

Retweet frequency is a parameter that indicates user interest in the topic of each tweet [37]. We also measured the number of favorites generated as an additional parameter of user interest, as previously shown [38]. Our results show that the Wake Up Weight Watchers hashtag obtained higher retweet and favorites frequencies than Eating Disorder Awareness Week hashtags and the control #eatingdisorder hashtag. Other Twitter metrics of diffusion (such as the potential impact and reach of tweets) were higher in Eating Disorder Awareness Week and Wake Up Weight Watchers tweets than in #eatingdisorder tweets. Furthermore, the awareness campaigns were associated with an increased number of contributors and tweets generated; there was an increased frequency of posting by very active users. However, these campaigns failed to promote the activity of very influential participants that were identified by the number of followers. It has been shown that verified accounts influence Twitter users [39].

### Strengths and Limitations

This study has some limitations. First, Twitter may not be reflective of the general population. Second, researchers cannot directly measure eating disorder behavior or clinical outcomes from tweets. Third, the codebook design and text analysis imply a degree of subjectivity. However, this methodology is consistent with previous medical research studies using Twitter and could be applied to different topics by different authors [40-42]. Furthermore, to address this issue, the study comprised a series of steps including the initial review, design of the codebook, and agreement between the coders. Although computerized machine learning methods have been tested to automatically identify and classify topics in medical research in social media [43], we used an analysis strategy based on the raters’ clinical expertise in mental health and endocrinology, which constitutes a qualitative advantage compared to automated strategies. On the other hand, it can be argued that #eatingdisorder content may be influenced by awareness campaigns. However, to our knowledge, there is no evidence of content seasonality. The time frame was selected according to awareness campaign dates. To collect data, we used the Twitter Firehose data stream, which is managed by Gnip and allows access to 100% of all public tweets that match a set of search criteria, which enables the creation of a very representative database. For the content analysis, we randomly collected a number of tweets that enabled us to make inferences from the text to summarize the content [44]. Finally, we did not determine whether a campaign prepared in advance (eg, Eating Disorder Awareness Week) affected Twitter activity differently (eg, user engagement) than a campaign that was more spontaneous (eg, Wake Up Weight Watchers).

### Conclusions

The initiatives Eating Disorder Awareness Week and Wake Up Weight Watchers increased the volume of eating disorder–related tweets, but the content of most tweets focused on raising awareness rather than promoting treatment-seeking behaviors, which would likely have a more significant outcome. Our findings support the need to reconsider the message and communication strategy employed by eating disorder awareness campaigns. The involvement of health institutions appears to be desirable because they are an important indicator of credibility and generated more tweets focused on medical information. Furthermore, the testimony of patients who have recovered from eating disorders should be promoted in these campaigns. Awareness campaigns may also wish to consider a strategy in which hashtags targeted toward those with eating disorders are included.

## Appendix

Multimedia Appendix 1 Potential reach and potential impact.

## Abbreviations

HAES: Health At Every Size
