# Supplementary material for: Eating Disorder Awareness Campaigns: Thematic and Quantitative Analysis Using Twitter
Source: J Med Internet Res. 2020 Jul 14;22(7):e17626. doi: 10.2196/17626 (PMC7388051; doi:10.2196/17626)
Supplement: Multimedia Appendix 1 [file jmir_v22i7e17626_app1.docx]

**Potential reach and potential impact**

We consider these metrics important because they provide a good approximation to how popular a certain hashtag is. The **impact** of a hashtag is defined by the amount of times that it may have been seen. The **impact of each** hashtag was calculated going user by user that participated in the hashtag multiplying the number of followers of each user by the number of tweets sent. Then, we add all these user´s results. *Each time that a user sends a tweet, he/she creates as many impressions of the hashtag we measure it as his/her number of followers.* The **reach**of a hashtag refers to how many Twitter users could have seen it. To calculate**the reach of each hashtag**we added all the followers of each Twitter user who participated in the hashtag. In this case the number of tweets that they posted is not relevant, only the number of Twitter followers. These metrics are analyzed by Tweet Binder, a Twitter tool that automatically tracks hashtags.

***Example***: #Event. We have 10 tweets sent by 2 users. These users are:

1. User Example @User1: it has 10 followers and sent 3 tweets with the hashtag #Event.
2. User Example @User2: it has 50 followers and sent 7 tweets with the hashtag #Event.

So we have 10 (3 + 7) tweets in total with the hashtag #Event. These tweets have been sent by 2 users. Let’s calculate now the **Twitter impacts** of each one of them:

1. User Example @User1: 10 followers and 3 tweets. Then we run this calculation: 10 followers x 3 tweets = 30 Twitter impressions or impacts.
2. User Example @User2: 50 followers and 7 tweets. We have to follow this rule to get the impacts: 50 followers x 7 tweets = 350 Twitter impressions or impacts.

That’s to say that the hashtag #Event’s impact is 380 impressions. We have to add the individual impacts created by each user. In this case is simple, we have 30 plus 350, so the total potential impact is 380.

***Example***: official hashtag #Event. We have 10 tweets sent by 2 users, but the number of tweets is not relevant:

1. User Example @User1: has 10 followers.
2. User Example @User2: has 50 followers.

In this case we will just add everyone’s followers and that’s how we get the global reach: 10 + 50 = 60 users reached. We have calculated the **Twitter reach.** So, if we combine both metrics impacts and reach, we could say that the hashtag #Event has generated 380 impacts and has reached 60 Twitter users.
